# Saunders’ Question Compends

**Published:** 1891-12

**Authors:** 


### Book Reviews.

SAUNDERS' QUESTION COMPENDS—Essentials of Physiology; Essentials of Anatomy. Philadelphia.

Both of the above Compends have lately been received by us, and having such authors as Dr. Hare and Dr. Wanerede, respectively, insures the completeness of the work. The plates in both works are among the best we have ever seen, and are themselves well worth the price of the books. C. D. R.

